# Novel XBP1s-independent function of IRE1 RNase in HIF-1α-mediated glycolysis upregulation in human macrophages upon stimulation with LPS or saturated fatty acid

**DOI:** 10.3389/fimmu.2023.1204126

**Published:** 2023-08-30

**Authors:** Margaud Iovino, Megan Colonval, Chloé Wilkin, Laurent L’homme, Cédric Lassence, Manon Campas, Olivier Peulen, Pascal de Tullio, Jacques Piette, Sylvie Legrand-Poels

**Affiliations:** ^1^ Laboratory of Immunometabolism and Nutrition, GIGA, ULiège, Liège, Belgium; ^2^ Univ. Lille, Inserm, CHU Lille, Institut Pasteur de Lille, U1011-EGID, Lille, France; ^3^ Laboratory of Virology and Immunology, GIGA, ULiège, Liège, Belgium; ^4^ Clinical Metabolomics Group, CIRM, ULiège, Liege, Belgium; ^5^ Metastasis Research Laboratory, GIGA, ULiège, Liège, Belgium

**Keywords:** saturated fatty acid, macrophages, glycolysis, HIF-1α, IRE1α, inflammation

## Abstract

In obesity, adipose tissue infiltrating macrophages acquire a unique pro-inflammatory polarization, thereby playing a key role in the development of chronic inflammation and Type 2 diabetes. Increased saturated fatty acids (SFAs) levels have been proposed to drive this specific polarization. Accordingly, we investigated the immunometabolic reprogramming in SFA-treated human macrophages. As expected, RNA sequencing highlighted a pro-inflammatory profile but also metabolic signatures including glycolysis and hypoxia as well as a strong unfolded protein response. Glycolysis upregulation was confirmed in SFA-treated macrophages by measuring glycolytic gene expression, glucose uptake, lactate production and extracellular acidification rate. Like in LPS-stimulated macrophages, glycolysis activation in SFA-treated macrophages was dependent on HIF-1α activation and fueled the production of pro-inflammatory cytokines. SFAs and LPS both induced IRE1α endoribonuclease activity, as demonstrated by *XBP1* mRNA splicing, but with different kinetics matching HIF-1α activation and the glycolytic gene expression. Interestingly, the knockdown of IRE1α and/or the pharmacological inhibition of its RNase activity prevented HIF-1α activation and significantly decreased glycolysis upregulation. Surprisingly, XBP1s appeared to be dispensable, as demonstrated by the lack of inhibiting effect of XBP1s knockdown on glycolytic genes expression, glucose uptake, lactate production and HIF-1α activation. These experiments demonstrate for the first time a key role of IRE1α in HIF-1α-mediated glycolysis upregulation in macrophages stimulated with pro-inflammatory triggers like LPS or SFAs through XBP1s-independent mechanism. IRE1 could mediate this novel function by targeting other transcripts (mRNA or pre-miRNA) through a mechanism called regulated IRE1-dependent decay or RIDD. Deciphering the underlying mechanisms of this novel IRE1 function might lead to novel therapeutic targets to curtail sterile obesity- or infection-linked inflammation.

## Introduction

The prevalence of overweight and obesity is increasing worldwide, and this is forecast to continue in the coming years. One in two adults in the USA are projected to have obesity by 2030, and about one in four to have severe obesity (1). Obesity promotes not only type 2 diabetes (T2D) and cardiovascular diseases, but also some cancers (2, 3). Metaflammation, an atypical metabolically-induced and chronic low-grade inflammation, is associated with obesity and plays an important role in the development of these comorbidities (4, 5). This one is initiated in visceral adipose tissue and is characterized by recruitment and activation of monocytes that differentiate into pro-inflammatory macrophages (4). However, these adipose tissue macrophages (ATMs), although they secrete pro-inflammatory cytokines (TNFα, IL-6, IL-1β), do not express the classical activation markers (M1) but present at their membrane alternative activation markers (M2) known to be involved in lipid metabolism (6). Interestingly, this phenotype of “Metabolically activated macrophages” (MMe) can be recapitulated by treating naive macrophages with long-chain saturated fatty acids (SFAs) like palmitate (C16:0) or stearate (C18:0) (6).

SFAs, unlike unsaturated fatty acids (UFAs), have long been recognized as pro-inflammatory mediators through Toll like receptor 4 (TLR4) activation (7–9). However, Lancaster et al. demonstrated very convincingly that palmitate does not bind to TLR4 (10). Instead, they showed that the palmitate-mediated c-jun N-terminal kinase (JNK) activation and pro-inflammatory cytokines expression require both palmitate uptake by macrophages and TLR4-dependent priming by endogenous TLR4 agonists present within serum (10). It is becoming increasingly clear that long chain SFAs mediate their impact after they are taken up by the cell and converted in acyl-coenzyme A, allowing them to be used in metabolic pathways such as lipid synthesis. Such events have been described upstream of the activation of the NLRP3 inflammasome by SFAs (11, 12). This intracellular protein complex assembles in response to homeostasis-altering molecular processes (HAMPs) and catalyzes the cleavage and maturation of the cytokines IL-1β and IL-18 (13). We and others have demonstrated that this complex is activated in ATMs of obese patients and diet induced obese (DIO) mice (14, 15) and that treatment of human and murine macrophages with SFAs recapitulates this activation (12, 16, 17). The incorporation of SFAs into membrane phospholipids followed by membrane stiffening has been proposed to trigger the loss of membrane homeostasis and the activation of the NLRP3 inflammasome (11, 12).

Such lipid bilayer stress can be at the origin of endoplasmic reticulum (ER) stress followed by unfolded protein response (UPR) (18, 19). Classical ER stress inducers like thapsigargin (Tg) and tunicamycin (Tm) induce an ER overload of unfolded/misfolded proteins that is sensed by the luminal domain of three ER-resident transmembrane proteins, PKR-like endoplasmic reticulum kinase (PERK), activating transcription factor 6 (ATF6), and inositol-requiring enzyme 1 (IRE1) (20, 21). Coordinated activation of these UPR sensors serves as the quality control mechanism to govern ER proteostasis and cope with ER stress (20, 21). Long-chain SFAs have been described to induce ER membrane disruption that is sensed by IRE1 and PERK transmembrane domain, leading to their oligomerization and activation (18, 19). By contrast, ATF6 is an ER stress sensor that has not been implicated in lipid sensing (11).

IRE1 regulates the most evolutionarily conserved arm of UPR (20, 21). In response to ER stress, IRE1 is activated through autophosphorylation, oligomerization and allosteric activation of its cytosolic RNase domain (20, 21). Once activated, IRE1 can induce three kinds of signaling pathways. IRE1 RNase activity promotes non canonical splicing of *XBP1* mRNA that produces the adaptive transcription factor XBP1s (20, 21). Sustained IRE1 RNase activity can also lead to the degradation of some mRNAs as well as certain pre-miRNAs through a process called regulated IRE1-dependent decay (RIDD) (22, 23). When chronically activated, phosphorylated IRE1 recruits TRAF2 to initiate JNK-mediated pro-apoptotic pathway and NF-κB-regulated pro-inflammatory signaling (24). IRE1 is involved in many biological processes in mammals, including cell survival/death determination, immunity and metabolism (25–28).

Although LPS does not elicit typical ER stress response, it specifically induces IRE1 activation by promoting its auto-phosphorylation through tumor necrosis factor (TNF) receptor-associated factor 6 (TRAF6)-dependent mechanism (29). This IRE1 activation was shown to regulate the TLR4/2-mediated pro-inflammatory activation of macrophages through the transcription factor XBP1s recruitment to the *IL6* and *TNF* gene promoters (30).

In this work, we highlight a new mechanism by which IRE1 RNase, independently of XBP1s, contributes to the pro-inflammatory polarization of macrophages upon stimulation with SFAs or LPS. This involves the activation of hypoxia-inducible factor 1-alpha (HIF-1α)-mediated aerobic glycolysis required to fuel the production of proinflammatory cytokines.

## Materials and methods

### Preparation of FFA solutions

The palmitic acid (C16:0, #P0500), stearic acid (C18:0, #S4751) and oleic acid (C18:1 #O1008) were purchased from Sigma (St Louis, USA). A 100 mM stock solution of sodium salt was prepared by dissolving fatty acids in 0.1 M NaOH. A 5% fatty acid free, low endotoxin BSA (#A8806, Sigma, St Louis, USA) solution was prepared in RPMI 1640. The FFA stock solution and the 5% BSA solution were mixed together to obtain a 2.5 mM working solution with FFA : BSA molar ratio at 3.4:1. After pH adjustment, the working solution was filtered through a 0.2 μm pore size membrane filter, aliquoted, and stored at -20°C for less than two months.

### Cell culture and treatments

Peripheral blood mononuclear cells (PBMCs) were purified by single step density gradient centrifugation with Ficoll-Paque PLUS (GE Healthcare, Chicago, USA) from buffy coat obtained from healthy donors after informed consent (Croix Rouge de Belgique). Monocytes were isolated from PBMCs using EasySep human CD14 positive selection kit II (StemCell) according to the manufacturer’s instructions. Monocyte-derived macrophages (MDMs) were generated by culturing freshly isolated monocytes (0.5, 1, 2 or 4x10^6^ monocytes/well for 24-, 12-, 6-well plate or 60 mm dish, respectively) in RPMI 1640 (Lonza, Basel, Switzerland) with 20% heat-inactivated FBS (Life Technologies Europe, 10270-106), 100 IU/mL penicillin (Lonza, Basel, Switzerland), 100 IU/mL streptomycin (Lonza, Basel, Switzerland) and 100 ng/mL of human M-CSF premium grade (Miltenyi Biotec, Bergisch Gladbach, Germany) for 7 days at 37°C under 5% CO2 atmosphere.

On the day prior to MDMs treatment, M-CSF-containing medium was replaced with new medium containing 10% FBS and 0.2% or 0.4% BSA (wt/vol) in experiments in which MDMs were to be treated with fatty acids 100µM or 200µM, respectively. On the day of MDMs treatments, this medium was replaced by RPMI + 10% FBS alone or supplemented with either BSA (0.2% or 0.4%) or FFA (100 µM or 200 µM) or LPS (Sigma Aldrich, SL2654, *Escherichia coli* 026:B6, 10 or 20 ng/ml) for various times according the experiment.

Appropriate vehicle or inhibitors, including STF-083010 (Sigma Aldrich, #SML0409), 4µ8C (Sigma Aldrich, #SML0949), trans-ISRIB (Cayman Chemicals, #16258), TAK242 (Sanbio) and 2-Deoxy-D-glucose (2-DG) (Sigma Aldrich) were added 1h before FFAs or LPS stimulation and maintained through the experiment.

### RNAseq

MDMs were treated by BSA, C18:0 and C18:1 (100 µM) for 3 and 16 hours. Total RNA was extracted using High pure RNA isolation kit (Roche) according to manufacturer’s instruction. RNA quantity was assessed using a spectrophotometer (NanoDrop Technologies). Total RNA integrity was evaluated by an Agilent 2100 Bioanalyser with a RNA 6000 Nano chip (Agilent Technologies) and all the samples had a RNA Integrity Number (RIN) ≥ 7.3. RNA libraries were prepared for each group of MDMs with the Truseq stranded mRNA sample prep kit from Illumina, based on polyA selection of mRNAs. cDNAs fragments were sequenced using the Illumina NextSeq500. Biological triplicates of the RNA Sequencing were performed for all the conditions.

Raw sequences were subjected to quality control analysis using FastQC (http://www.bioinformatics.babraham.ac.uk/projects/fastqc/). The reads for each condition were mapped the human reference genome hg19 from UCSC using TopHat2 version 2.0.7 and stored as BAM files. Differential expression values were evaluated using Cufflinks 2.1.1 package (31). The three MDM replicates treated with C18:0 belonging to the different timepoints (3 hours and 16 hours) were tested for differential expression versus the control condition (BSA 3 hours and 16 hours respectively). Genes with a q value below 0.05 were considered as significant for differential expression.

### RT-qPCR analysis

Total RNAs were extracted with RNeasy Mini Kit (Qiagen) according to the manufacturer’s recommendations. Purified RNAs were reverse-transcribed to complementary DNA (cDNA) by using the RevertAid H Minus First Strand cDNA Synthesis Kit (Thermo Scientific). qPCR was performed by using FastStart Universal SYBR Green Master Mix (Roche) and ran on a LightCycler 480 (Roche Applied Science, Penzberg, Germany). Gene expressions were calculated using the 2^-ΔΔCT method. TBPH (TAR Binding-Protein) was chosen as housekeeping gene. Primers were designed with Primer-BLAST (National Center for Biotechnology Information (NCBI)) to amplify all the isoforms of the target gene, except for XBP1-s primers that were designed to only amplify the spliced isoform (isoform 2, NM_001079539). Primer sequences are provided in **Supplementary Table S1**.

### Glucose and lactate measurements

The NMR spectra were recorded at 298 K on a Bruker Neo spectrometer (Bruker) operating at 500.13 MHz for proton and equipped with a TCI cryoprobe (Bruker). Deuterium oxide (99.96% D) and trimethylsilyl-3-propionic acid-d4 (TMSP) were purchased from Eurisotop (St-Aubin, France), phosphate buffer powder 0.1 M and calcium formate were purchased from Sigma-Aldrich. Deuterated solvents were used as the internal lock. The data have been processed with Bruker TOSPIN 4.1 (Bruker) software with standard parameter set. Phase and baseline correction were performed manually over the entire range of the spectra and the δ scale was calibrated to 0 ppm using the internal standard TMSP. For extracellular lactate and glucose dosage, 400 μl of collected culture media supernatants were supplemented with 47 μl of deuterated phosphate buffer (pH 7.4), 24 μl of a 10 mM deuterated solution of calcium formate and 70 μl of a 5mM deuterated solution of TMSP. The solution was distributed into 5-mm tubes for NMR measurement. 1H NMR spectra were acquired using a 1D NOESY sequence with presaturation and 64 transients and 4 dummy scans. Based on the formate peak (8.46 ppm), lactate and glucose concentration were then obtained by using the Chenomx NMR Suite software (version 9.02, Edmonton, CA).

### 2-NBDG uptake

The fluorescent glucose analogue 2-(N-(7-nitrobenz-2-oxa-1,3-diazol-4-yl)amino)-2-deoxyglucose (2-NBDG, Invitrogen) was used to measure glucose uptake by MDMs. After treatment of the macrophages for various times, the medium was replaced by RPMI 1640 without glucose supplemented with 2-NBDG (30µM). The MDMs were maintained in culture for 30 min before being gently scrapped with EDTA (10mM, pH 6.14). Following two PBS washes, labeling with 7-aminoactinomycin D (7-AAD, Thermo Scientific) was carried out according to the company’s instructions in order to assess cell viability. Results are acquired on a FACS Verse flow cytometer (Becton Dickinson) and analyzed using FlowJo 9.3.2 software (Tree Star Inc., 645 Ashland, OR).

### Extracellular flux analysis

Real time bioenergetic profiles of MDMs were obtained by measuring oxygen consumption rate (OCR – pmol/min) and extracellular acidification rate (ECAR – mpH/min) using a Seahorse XFp extracellular flux analyzer (Agilent, Santa Clara, CA, USA). Monocytes were differentiated in MDMs for 7 days in XFp mini-plates (Agilent). MDMs were left untreated or treated with LPS (20 ng/ml) for 16 h, or with BSA and C18:0 (200µM) for 24h. After treatments, cells were kept in unbuffered serum-free DMEM (Basal DMEM) supplemented with glutamine (2mM), pH 7.4 at 37°C, and ambient CO2 for 1 h before the assay. During the assay, cells were successively treated with glucose (10mM), oligomycin (1µM), FCCP (2µM) and rotenone/antimycin A mix (0.5µM each). OCR and ECAR were normalized according to cell number evaluated by Hoechst incorporation (arbitrary unit – A.U.).

### ELISA

IL-1 beta, IL-6 and IL-8 human uncoated ELISA Kits (ThermoFisher) were used to measure secreted cytokines in supernatants according to the manufacturer’s recommendations.

### Western blot analysis

Cells were lysed in total lysis buffer (50 mM Tris-HCl at pH 8, 150mM NaCl, 5mM EDTA, 1% NP-40, 0.5% Na-deoxycholate, SDS 0.1%, 1mM PMSF and complete protease inhibitor cocktail (Roche)) and subjected to SDS-PAGE. For the preparation of nuclear extracts, approximately 4x10^6^ cells (dish 60 mm) were scrapped in the hypotonic buffer 1 [10 mM HEPES-KOH pH 7.9, 10 mM KCl, 0.5% Igepal, 0.1 mM EDTA, 1 mM DTT, 1 mM PMSF and complete protease inhibitor cocktail (Roche)] and incubated for 10 min at 4°C. Samples were centrifuged and nuclei pellets were incubated for 30 min in the hypertonic buffer 2 (50 mM HEPES-KOH pH 7.9, 50 mM KCl, 400 mM NaCl, 10% glycerol, 0.1 mM EDTA, 1 mM PMSF, complete protease inhibitor cocktail). After centrifugation for 30 min, the supernatants containing nuclear proteins were harvested.

The following primary antibodies were used: anti-HIF-1α (Cell Signaling Technology, #36169), anti-XBP1s (Cell Signaling Technology, #12782), anti-GLUT1 (Cell Signaling Technology, #12939), anti-IRE1α (Cell Signaling Technology, #3294), anti-RNA polymerase II (Santa Cruz, sc-899), anti-HSP90 (Cell Signaling Technology, #4877s) and anti-GAPDH (Thermo Ambion, #AM4300). The secondary antibodies used for the revelation were HRP-linked anti-rabbit or anti-mouse IgG (Cell Signaling Technology, #7074 or #7076). For chemiluminescent western blot, revelation was performed with ECL (Pierce, Waltham, USA) by using the digital imaging system ImageQuant LAS 4000 (GE Healthcare, Chicago, USA) and quantification achieved with the ImageQuant TL software (version 7.0, GE Healthcare, Chicago, USA) or the ImageJ 2.0.0 software.

### siRNA transfection

MDMs were transfected by using lipofectamine RNAiMax (13778, Invitrogen) according to manufacturer’s instructions. Predesigned siRNA targeting human HIF-1α, XBP-1s and IRE1α mRNA or control (CT) siRNA targeting any sequence were purchased from Integrated DNA Technologies (IDT, Coralville, USA) (TriFECTa DsiRNA Kit) (**Table S2**). MDMs were transfected with siRNA XBP1s and siRNA HIF-1α for 24h and siRNA IRE1α for 48h before SFAs or LPS stimulation.

### Statistical analyses

All statistical analyses were carried out using GraphPad Prism 7 for Windows (GraphPad Software, Inc., San Diego, USA) and presented as the means ± standard deviation (SD). When one independent variable was involved, two-tailed Student’s t-test was performed to compare two groups and one-way ANOVA with Dunnett’s multiple comparisons test to compare more than two groups. When two independent variables were involved, two-way ANOVA was used with Sidak’s or Tukey’s multiple comparisons test. The statistical test used and the number of biological replicates (n) are described in each figure legend.

## Results

### Transcriptome of C18:0-treated human macrophages highlights both glycolysis and hypoxia signatures

To assess the phenotype of macrophages maintained in FFA-rich environment, we sequenced the whole transcriptome of monocytes-derived macrophages (MDMs) treated with saturated or unsaturated fatty acid or with vehicle (BSA) for a short (3h) or extended period (16h). Stearate (C18:0) was chosen because it is the second most abundant SFA in tissues and blood after palmitate (C16:0) and has been shown to be more effective than palmitate in inducing cytokine production at low concentrations in our previous studies (12, 17). The low cytotoxic concentration of 100 μM was chosen for both C18:0 and the corresponding UFA, C18:1 or oleate.

**Figure 1:**
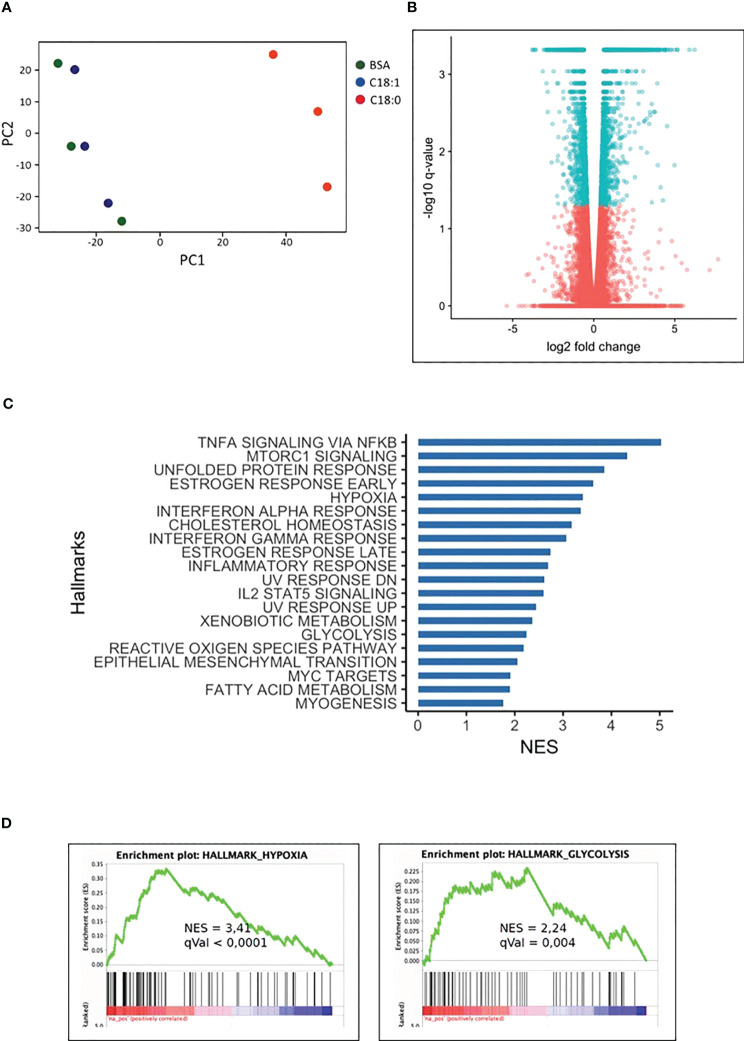
C18:0-treated human macrophages transcriptome. MDMs were treated for 16 hours with BSA, C18:0 and C18:1 at 100µM. **(A)** Principal component analysis (PCA) was performed using normalized RNA-Seq data of all the genes obtained with the three different treatments of MDMs coming from 3 independent buffy coats. **(B)** Volcano plot representation of differential expression analysis of genes in C18:0-treated MDMs. Blue points mark the genes with significantly increased or decreased (q value < 0,05) expression and pink points correspond to genes with no significantly different expression in C18:0-treated MDMs compared to BSA-treated MDMs. **(C)** Top 20 of upregulated “Hallmark pathways” in C18:0-treated MDMs vs BSA-treated MDMs. Gene set enrichment analysis (GSEA) was made with genes differentially expressed by MDMs treated for 16 hours with C18:0 100µM compared to BSA 100µM and having a q value smaller than 0.05 (NES = normalized enrichment score). **(D)** GSEA plots showing enrichment of hypoxia and glycolysis hallmark pathways in C18:0-treated MDMs with their NES and q values.

The principal component analysis (**Figure 1A**) shows that C18:0, unlike C18:1, induces a strong MDMs transcriptome modulation after 16 hours; 1482 and 1861 genes are down- and up-regulated, respectively, compared to control MDMs (BSA) (**Figure 1B**). No significant change in the transcriptome has been observed after 3 h of treatment with C18:0 compared to BSA (data not shown). Gene set enrichment analysis (GSEA) was performed on differentially upregulated genes in C18:0 (16h)-treated MDMs. There were 20 hallmark gene sets upregulated with a q-value ≤ 0.05 (**Figure 1C**). As expected, GSEA highlighted the pro-inflammatory profile of SFA-treated macrophages as well as a strong unfolded protein response (UPR). This analysis also allowed to reveal metabolic signatures like glycolysis and hypoxia (**Figure 1D**).

The pro-inflammatory activation and return to homeostasis of macrophages are intimately linked and dependent on dynamic changes in cellular metabolism; an increased aerobic glycolysis is notably required to fuel the LPS-mediated pro-inflammatory polarization of macrophages (32). Accordingly, we wanted to investigate whether C18:0-treated MDMs are also subject to glycolysis activation and whether this is involved in their pro-inflammatory polarization.

### Like in LPS-challenged MDMs, glycolysis is preferentially adopted by human macrophages upon activation with C18:0

Almost all studies using exogenous fatty acids solutions take BSA to conjugate fatty acids. However, it is now well accepted that BSA-associated contaminants may activate some TLR4-mediated signaling pathways (10, 32–35). To minimize such potentially confounding effects, differentiated MDMs were cultured overnight in medium containing FBS (10%) and BSA (*i.e.*, the medium used for treating MDMs, but without fatty acids) prior to treatment with either SFA or vehicle (BSA). This pre-treatment induces TLR4 tolerance that can be described as a transient state of altered responsiveness of cells to the repeated or chronic activation of TLRs (36). In this way, MDMs are tolerant when we apply the second treatment with SFAs, which allows us to dissociate the impact of SFAs from this of BSA and to investigate the response to SFAs per se.

**Figure 2:**
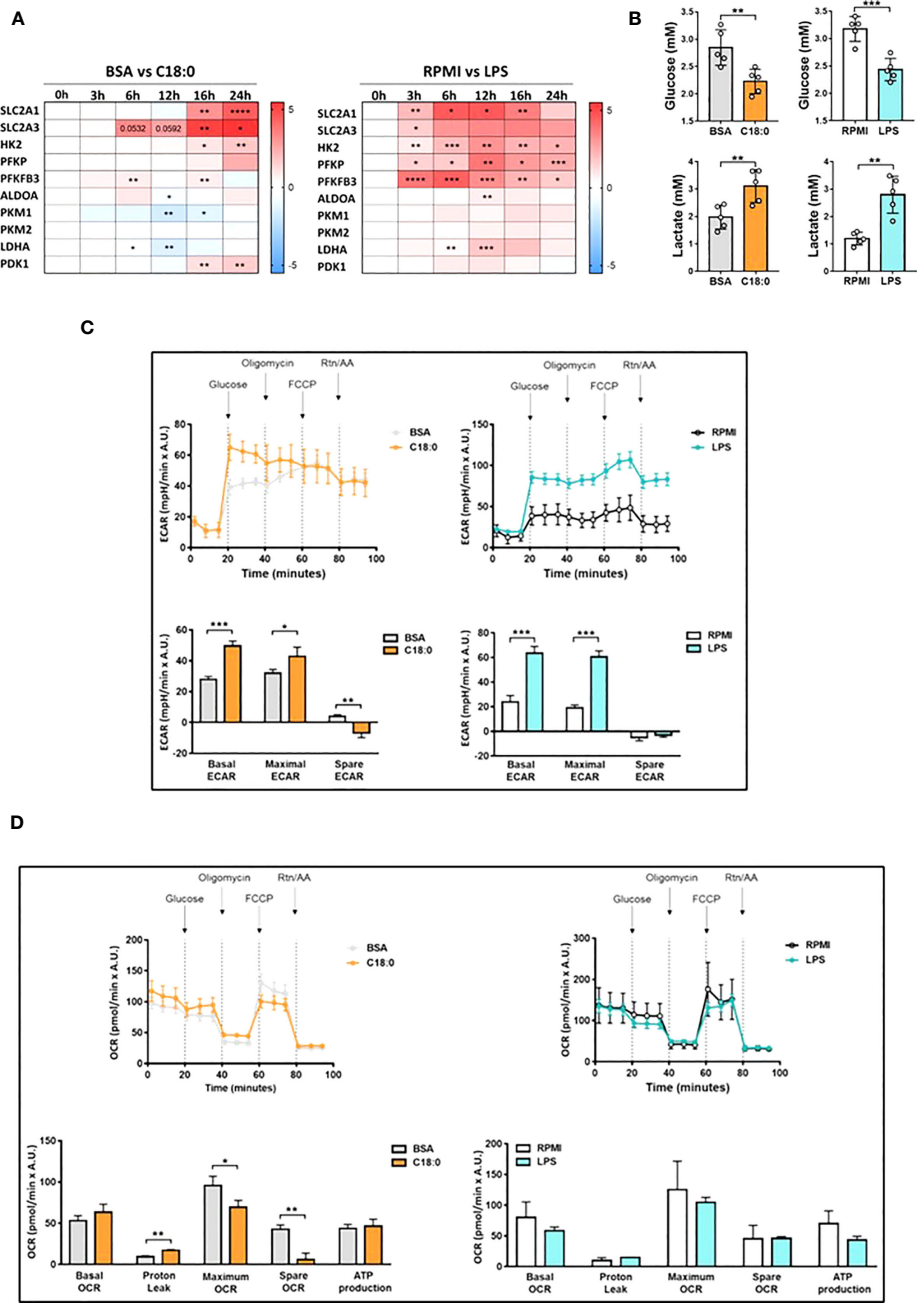
Upregulation of aerobic glycolysis in C18:0- and LPS-treated human macrophages. **(A)** Heatmap depicting Log2 fold change of glycolytic genes determined by qRT-PCR experiments in MDMs cultured with C18:0 (100µM) or LPS (10 ng/ml) for indicated times compared to MDMs cultured with BSA or medium alone (RPMI), respectively (n=4). Data are mean ± SD. Unpaired t-test. *p<0.05, **p<0.01, ***p<0.001, ****p<0.0001. **(B)** Glucose and lactate concentrations in supernatants of MDMs treated with BSA, C18:0 (100 µM), LPS (10 ng/ml) or maintained in medium alone (RPMI) for 24h. Extracellular glucose and lactate concentrations were measured by NMR (n=5). Data are mean ± SD. Unpaired t-test. **p<0.01, ***p<0.001. **(C, D)** Seahorse analysis of extracellular acidification rate (ECAR) **(C)** and oxygen-consumption rate (OCR) **(D)** on MDMs treated with BSA and C18:0 (200 µM) for 24h or maintained in medium alone (RPMI) and treated with LPS (20 ng/ml) for 16h. ECAR and OCR were normalized according to Hoechst incorporation (A.U.). Data are mean ± SD from three technical replicates for each condition. Statistical analysis of basal and maximal ECAR, maximum and spare OCR and OCR-related proton leak was performed by Student’s t-test. *p < 0.05, **p < 0.01, ***p < 0.001.

First, we wanted to confirm the upregulation of genes involved in glycolysis by qRT-PCR experiments in C18:0-treated MDMs in comparison with LPS-stimulated MDMs. The heatmap of the **Figure 2A** shows the fold change (log2 FC) induced by C18:0 or LPS for each gene relative to the respective control, BSA or RPMI. C18:0 treatment induces a significant upregulation of both genes *(SLC2A1* and *SLC2A3*) encoding for glucose transporters, GLUT1 and GLUT3, respectively, as well as those encoding for the two first rate-limiting enzymes involved in the glycolytic pathway, Hexokinase II (HK2) and phosphofructokinase (PFKP). The C18:0-mediated activation kinetics are slow with no visible induction before 16h of treatment while the LPS upregulating effect is already observed after 3h, shows a peak between 6 and 12 hours and then gradually decreases. Another difference between C18:0 and LPS is the lack of stimulatory effect of C18:0 on the expression of genes encoding downstream enzymes such as the aldolase A (ALDOA), the two isoforms of pyruvate kinase (PKM1 and PKM2) and the lactate dehydrogenase A (LDHA). Interestingly, C18:0 induces the expression of the pyruvate dehydrogenase kinase 1 (PDK1) that phosphorylates and inhibits the pyruvate dehydrogenase and, thereby, prevents the pyruvate from entering the mitochondria and favors the glycolytic switch.

The glucose consumption and lactate production were assessed by comparing extracellular concentrations of glucose and lactate between C18:0- and BSA-treated MDMs or between MDMs stimulated with LPS and those maintained in medium alone for 24h. Both glucose and lactate concentrations were measured by NMR. The **Figure 2B** shows that C18:0 and LPS treatments induce both a significant decrease of glucose levels and significant increase of lactate concentrations compared to their respective control, suggesting an increased aerobic glycolysis. As expected, MDMs treatment with palmitate (C16:0) also stimulates both glucose uptake and lactate secretion at similar extent than C18:0 (**Figure S1A**). Increased glucose consumption was also validated by measuring the uptake of 2NBDG, a fluorescent glucose analog (**Figure S1B**).

To further investigate the impact of SFAs on macrophage bioenergetic metabolism, we performed extracellular flux analysis with the Seahorse XFp-analyzer. As expected, MDMs challenging with LPS results in greater extracellular acidification rate (ECAR), an index of glycolysis (**Figure 2C**). However, as already reported (37), resting and LPS-stimulated human MDMs, unlike murine bone marrow-derived macrophages (BMDMs), do not show any glycolytic reserve (spare-ECAR) when treated with oligomycin that blocks oxidative phosphorylation (OxPhos) and allows maximal glycolysis. C18:0 treatment also significantly upregulates ECAR in MDMs but induces a significant decrease of the glycolytic reserve compared to BSA (**Figure 2C**). As previously reported (38), neither basal oxygen consumption rate (OCR) nor maximal OCR induced upon uncoupling the respiratory chain with FCCP are affected by LPS treatment in human MDMs (**Figure 2D**) while LPS challenge is known to downregulate both basal and maximal OCR in BMDMs. The OCR profile of C18:0-stimulated MDMs shows no effect on basal OCR but demonstrates a significant decrease in maximal OCR compared to BSA-treated MDMs. The spare OCR capacity is greatly reduced, the oxygen consumption associated with proton leak is significantly increased while OCR-linked ATP production remains unchanged compared to MDMs treated with BSA (**Figure 2D**).

Altogether, these data suggest that, like in LPS-stimulated human MDMs, glycolysis is preferentially adopted by human macrophages upon activation with C18:0.

### Glycolysis fuels the production of pro-inflammatory cytokines by human macrophages upon activation with C18:0

**Figure 3:**
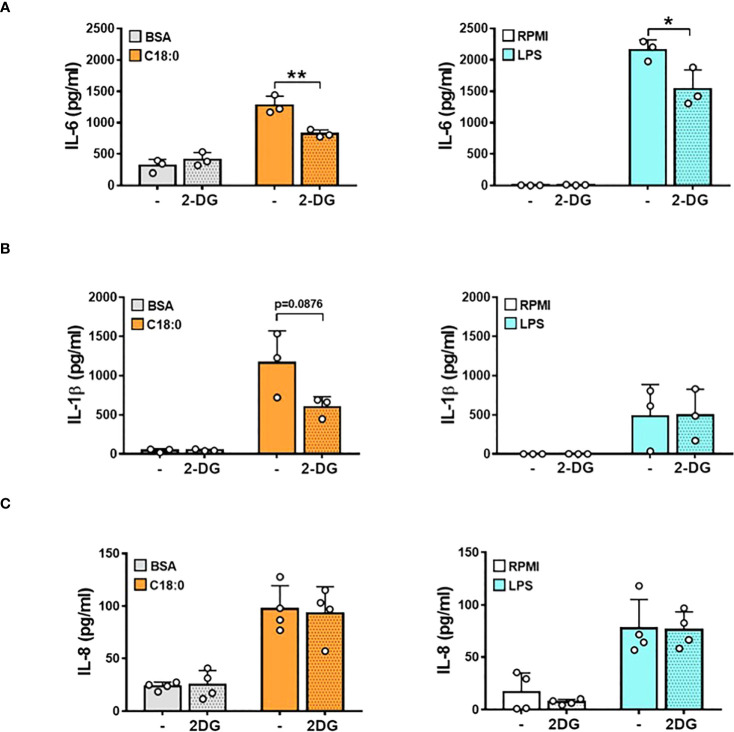
Glycolysis fuels the production of pro-inflammatory cytokines in C18:0- and LPS-stimulated human macrophages. MDMs were pre-treated or not with 2-DG (10 mM) for 1h before being stimulated with BSA, C18:0 (100 µM), LPS (10 ng/ml) or maintained in RPMI alone for 24h. The concentrations of IL-6 **(A)**, IL-1β **(B)** and IL-8 **(C)** were measured in the supernatants by ELISA. Data are mean ± SD. Unpaired t-test (n=3). *p < 0.05, **p < 0.01.

To investigate whether this C18:0-induced metabolic reprogramming promotes pro-inflammatory cytokines release, we tested the effect of 2-deoxy glucose (2-DG), a competitive glycolysis inhibitor, on IL-6, IL-1β and IL-8 secretion (**Figure 3**). As expected, LPS challenging of MDMs induces an important IL-6 secretion that is partially, but significantly, decreased by 2-DG pretreatment (**Figure 3A**). MDMs cultured with BSA produce significant IL-6 levels that are not affected by 2-DG addition. Treatment with C18:0 further stimulates IL-6 secretion that is significantly reduced in the presence of 2-DG suggesting that glycolysis controls IL-6 release in response to C18:0.

As shown in **Figure 3B**, LPS treatment leads to a moderate IL-1β release compared to C18:0. This result can be explained by the fact that IL-1β release requires two signals; (1) a first priming signal leading to *IL1B* gene transcription and IL-1β precursor (pro-IL1β) synthesis mediated by TLR agonists like LPS and (2) a second signal triggered by homeostasis-altering molecular processes (HAMPs) or Danger-Associated Molecular Patterns (DAMPs) to activate NLRP3 inflammasome-mediated pro-IL-1β processing and mature IL-1β release (13). In LPS-treated MDMs, a small fraction of *de novo* synthesized pro-IL-1β is probably cleaved by a weak constitutive activity of caspase 1 (39), leading to the secretion of low IL-1β levels as observed in **Figure 3B**. We previously demonstrated that SFAs are able to induce NLRP3 inflammasome activation in human monocytes and macrophages (12, 17). In this study, the BSA priming of MDMs probably induces the moderate synthesis of the pro-IL-1β that is efficiently cleaved by C18:0-stimulated NLRP3 inflammasome, as demonstrated by significant levels of secreted IL-1β (**Figure 3B**). While inhibiting glycolysis by 2-DG has no effect on LPS-induced IL-1β levels, this treatment tends to decrease IL-1β secretion in response to C18:0 probably by impacting NLRP3 inflammasome activation as already reported (40). The treatment with 2-DG has no effect on LPS- and C18:0-induced IL-8 production (**Figure 3C**), excluding the hypothesis that the inhibiting effect of 2-DG on IL-6 and IL-1β release could be due to a cytotoxic effect.

### HIF-1α is involved in the upregulation of the glycolysis in C18:0- and LPS-treated human macrophages

In a resting cell, HIF-1α is hydroxylated at conserved proline residues by the prolyl hydroxylases (PHDs) (41). This hydroxylation targets HIF-1α for ubiquitination and rapid proteasomal degradation. The PHDs are oxygen dependent; thus, under normoxic conditions, HIF-1α is continuously turned over, resulting in low basal HIF-1α levels. In hypoxic conditions, PHDs are inhibited and HIF-1α can accumulate and form a heterodimeric complex with HIF-1β that translocates to the nucleus and increases transcription of hypoxia response elements (HRE)-containing genes (41). HIF target genes are involved in cellular adaptation to hypoxia, metabolism, and cell function (41). In addition to be implicated in adaptation to environmental changes, HIF pathway has been linked to the key metabolic changes in innate immune cells in response to pattern recognition receptor (PRR) ligation (41). In LPS-treated macrophages, HIF-1α drives the glycolytic switch by inducing the expression of several genes including *SLC2A1*, *HK2*, *PFKP* and *PDK1*.

**Figure 4:**
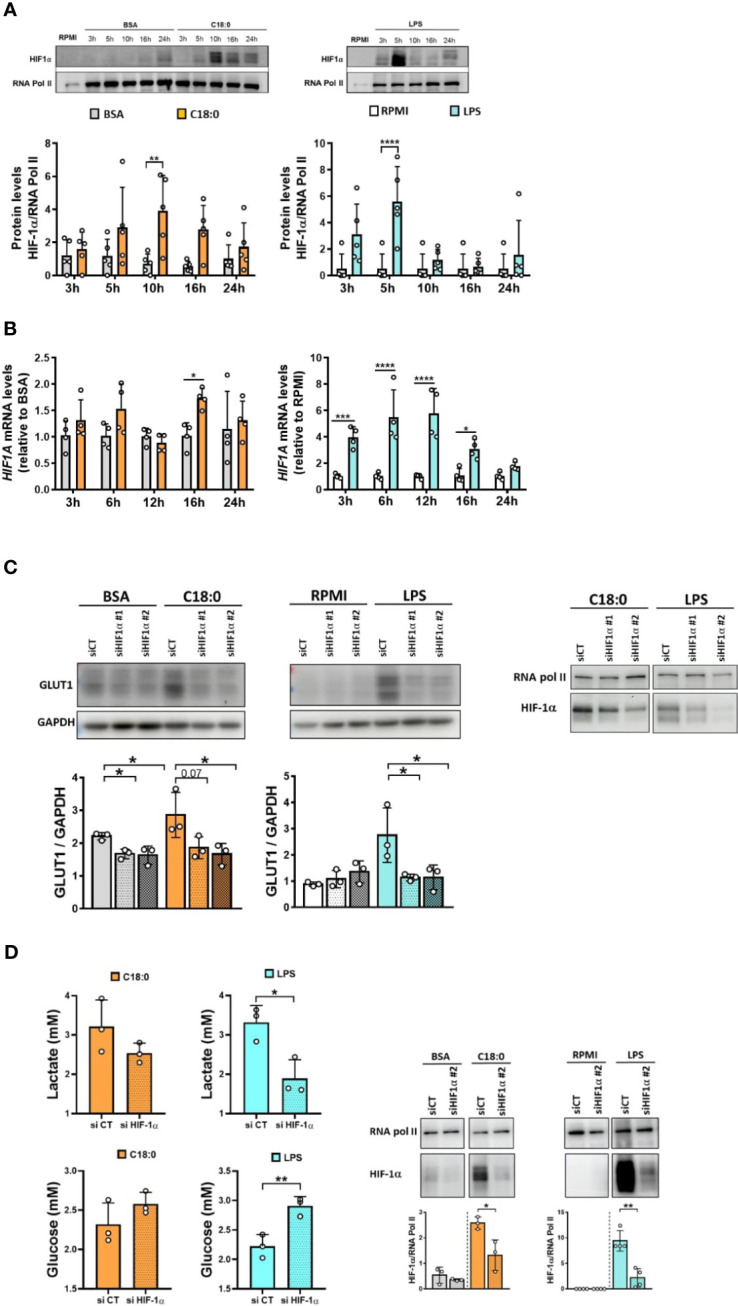
HIF-1α is involved in C18:0- and LPS-mediated glycolysis activation. **(A, B)** MDMs were treated with BSA, C18:0 (100 µM), LPS (10 ng/ml) or maintained in RPMI alone for indicated times. **(A)** Western Blot analysis of HIF-1α and RNA polymerase II protein levels in nuclear extracts (n=5). **(B)** qRT-PCR analysis of *HIF1A* mRNA levels (n=4). Data are mean ± SD. Two-Way ANOVA, Sidak’s multiple comparisons test, *p < 0.05, **p < 0.01, ***p < 0.001, ****p<0.0001. **(C)** MDMs were transfected with siRNA control (CT) or siRNA targeting HIF-1α (siRNA#1 or siRNA#2) 24h before treating them or not (RPMI) with BSA, C18:0 (100 µM) or LPS (10 ng/ml). On the left: Western Blot analysis of GLUT1 and GAPDH protein levels in total extracts after 24h treatment (n=3). One-way ANOVA with Dunnett’s multiple comparisons test. On the right: Western Blot analysis of HIF-1α and RNA polymerase II protein levels in nuclear extracts after treatment for 10h (C18:0) or 5h (LPS) (one representative experiment). **(D)** MDMs were transfected with siRNA control (CT) or siRNA targeting HIF-1α (siRNA#2) 24h before treating them or not (RPMI) with BSA, C18:0 (100 µM) or LPS (10 ng/ml). On the left: Lactate and glucose concentrations were measured by NMR in the supernatants of MDMs treated for 24h (n=3). On the right: Western Blot analysis of HIF-1α and RNA polymerase II protein levels in nuclear extracts after treatment for 10h (BSA, C18:0) or 5h (RPMI, LPS) (n=3). Unpaired t-test *p < 0.05, **p < 0.01.

To investigate whether C18:0 treatment also leads to HIF-1α activation, we proceeded to the detection of HIF-1α in the nuclear extracts of BSA- and C18:0 -treated MDMs. We used LPS-stimulated MDMs as positive controls. As expected, LPS induces a strong, rapid and transient HIF-1α accumulation in the nucleus, with a peak at 5 hours (**Figure 4A**). C18:0 is also able to drive HIF-1α activation that is much slower (peak at 10h) and extended until 24h (**Figure 4A**). In addition to post-transcriptional activation of HIF-1α, LPS also induces upregulation of *HIF1A* mRNA as previously described (42) unlike C18:0 which has very little impact on *HIF1A* mRNA levels relative to BSA control (**Figure 4B**).

To confirm the role of HIF-1α in the glycolytic switch, we performed the knockdown of HIF-1α with two different siRNA and study the impact on GLUT1 protein levels in total cellular extracts. The depletion of HIF-1α in nuclear extracts of MDMs after siRNA transfection and treatments is shown in the **Figure 4C** (right panel). The knockdown of HIF-1α by both siRNA prevents GLUT1 upregulation in response to both C18:0 and LPS (**Figure 4C**, left panel). The glucose uptake and lactate secretion by C18:0- and LPS-treated MDMs are also affected by the knockdown of HIF-1α (**Figure 4D**, left panel). However, the effect is not significant for C18:0, maybe because the silencing of HIF-1α is less pronounced (**Figure 4D**, right panel).

### Involvement of IRE1’s RNase activity in both C18:0- and LPS-induced glycolytic switch

**Figure 5:**
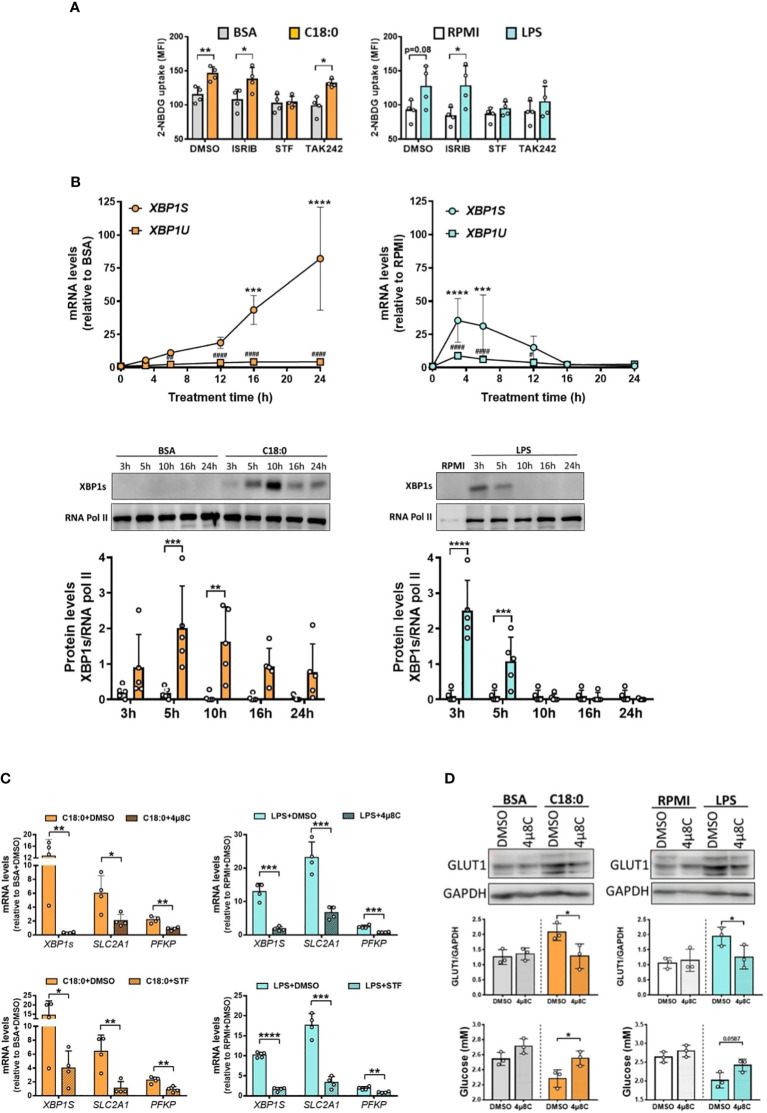
IRE1’s RNase activity is involved in C18:0- and LPS-mediated glycolysis activation. **(A)** MDMs were pre-treated for 1h with ISRIB (10nM), STF (100µM), TAK 242 (1µM) or vehicle (DMSO) before the addition or not (RPMI) of BSA, C18:0 (100 µM) or LPS (10 ng/ml). After 24h, glucose uptake was determined by the fluorescence of 2-NBDG (n=4). Data are mean ± SD. Unpaired t-test *p < 0.05, **p < 0.01. **(B)** MDMs were treated with BSA, C18:0 (100 µM), LPS (10 ng/ml) or maintained in medium (RPMI) alone for indicated times. qRT-PCR analysis of unspliced (*XBP1U*) and spliced (*XBP1S*) *XBP1* mRNA levels (top panel) and western blot analysis of XBP1s and RNA polymerase II protein levels in nuclear extracts (middle and bottom panels). Two-Way ANOVA, Sidak’s multiple comparisons test; *XBP1S* mRNA or XBP1s protein (C18:0 or LPS vs BSA or RPMI, respectively), ****p< 0.0001, ***p< 0.001, **p<0.01, *p<0.05 and *XBP1U* mRNA (C18:0 or LPS vs BSA or RPMI, respectively), ^###^p<0.001, ^##^p<0.01, ^#^p<0.05. **(C)** MDMs were pre-treated for 1h with STF (100 µM), 4µ8C (50 µM) or vehicule (DMSO) before the addition or not (RPMI) of BSA and C18:0 (100 µM) for 24h or LPS (10 ng/ml) for 6h. qRT-PCR analysis of *XBP1S*, *SLC2A1* and *PFKP* mRNA levels. Unpaired t-test, ****p< 0.0001, ***p< 0.001, **p<0.01, *p<0.05. **(D)** MDMs were pre-treated for 1h with 4µ8C (50 µM) or vehicule (DMSO) before the addition or not (RPMI) of BSA, C18:0 (100 µM) or LPS (10 ng/ml) for 24h. Western blot analysis of GLUT1 and GAPDH protein levels in total extracts (Top and middle panels) and determination of extracellular glucose concentrations by NMR (bottom panel). Unpaired t-test, *p<0.05.

The transcriptome of human macrophages treated with stearate reveals a strong UPR signature (**Figure 1C**). We have previously shown a significant activation of both UPR branches involving IRE1 and PERK sensors in MDMs treated with C18:0 (12, 17). To test the potential role of both these pathways in C18:0-mediated glycolytic switch, we first used two pharmacological inhibitors, STF and ISRIB (integrated stress response inhibitor), previously reported to efficiently target the IRE1’s RNase activity and the eIF2α phosphorylation by PERK, respectively, in C18:0-treated MDMs (12, 17). A specific inhibitor of both TLR4-induced MyD88 and TRIF-dependent pathways [TAK-242 (43)] was also used. The impact of all inhibitors was first tested on 2NBDG uptake induced by C18:0 and LPS. As expected, TAK242 prevents LPS but not C18:0 to upregulate the glucose uptake (**Figure 5A**). Interestingly, while the inhibition of the PERK-eIF2α pathway by ISRIB has no effect, the inhibition of IRE1’s RNase activity by STF completely prevents the stimulation of the glucose uptake in response to both C18:0 and LPS (**Figure 5A**).

Since the IRE1 RNase activity seems to be required for both C18:0- and LPS-stimulated glucose uptake, we investigated whether the IRE1 activation kinetics in response to C18:0 and LPS fit with both HIF-1α and glycolysis activation. IRE1 activation was assessed by measuring both spliced *XBP1S* mRNA and nuclear XBP1s protein levels. Again, the accumulation of the *XBP1S* mRNA is rapid and transient in response to LPS with a peak at 3h while the splicing is much slower and sustained until 24h in the case of C18:0 (**Figure 5B**, top panel). A slight upregulation of unspliced mRNA (*XBP1U*) levels is also observed but this alone cannot explain the large increase in spliced mRNA levels (**Figure 5B**, top panel). Once translated, the XBP1s protein translocates into the nucleus. As expected, its appearance in nuclei is rapid and very transient in the case of LPS. In the C18:0-treated MDMs, the translocation of XBP1s is slower, reaches its peak between 5h and 10h then decreases but XBP1s is still detectable in the nuclei after 24h (**Figure 5B**, middle and bottom panels). Interestingly, the fate of IRE1α protein in macrophages during LPS challenge is completely different than after activation with C18:0. In resting macrophages, the levels of IRE1α protein are low (**Figure S2A**). Upon activation with C18:0, these levels begin to gradually increase from time 5h and are maintained until at least 24h, while two induction waves are observed for LPS, the first at a short time (3h) and the second later (16 to 24h) (**Figure S2A**).

Altogether, these data demonstrate that each inducer, C18:0 or LPS, activates the three pathways, IRE1-XBP1s, HIF-1α and glycolysis, with matching kinetics. Both transcription factors, HIF-1α and XBP1s, are therefore found simultaneously in the nucleus. Knowing that XBP1s has already been reported as a co-activator of HIF-1α in the transcription of the gene encoding GLUT1 (*SLC2A1*) in breast cancer cells (44), it was tempting to speculate such a role in our model of proinflammatory macrophages.

To investigate whether the IRE1-XBP1s pathway is involved in the upregulation of glycolysis genes, we studied the impact of both inhibitors of IRE1’s RNase activity (STF and 4µ8C) on their expression in MDMs stimulated with LPS or C18:0. **Figure 5C** confirms that both drugs are able to inhibit the splicing of *XBP1* mRNA as demonstrated by the significant decrease of the *XBP1S* mRNA levels in both LPS- and C18:0-treated MDMs. The levels of *SLC2A1* and *PFKP* mRNA are also significantly downregulated by both inhibitors suggesting a role of IRE1’s RNase activity in the expression of these genes in response to LPS and C18:0 (**Figure 5C**). The effect of STF and 4µ8C was also investigated on the expression of *SLC2A3*, *HK2* and *PDK1* genes known to be involved in glycolytic shift and previously reported to be also upregulated in C18:0- and LPS-treated MDMs (**Figure 2A**). Both drugs exert an inhibiting effect on *PDK1* but not *SLC2A3* expression (**Figure S2B**). Surprisingly, LPS-mediated *HK2* gene upregulation is significantly impacted by both inhibitors while no effect is observed in the case of C18:0 (**Figure S2B**). The role of IRE1’s RNase activity in glycolysis upregulation was also confirmed by showing that 4µ8C reverses the increase of both GLUT1 protein expression (**Figure 5D**, top and middle panels) and glucose uptake in response to both LPS and C18:0 (**Figure 5D**, bottom panel). 

### XBP1s is dispensable in both C18:0- and LPS-induced glycolytic switch

**Figure 6:**
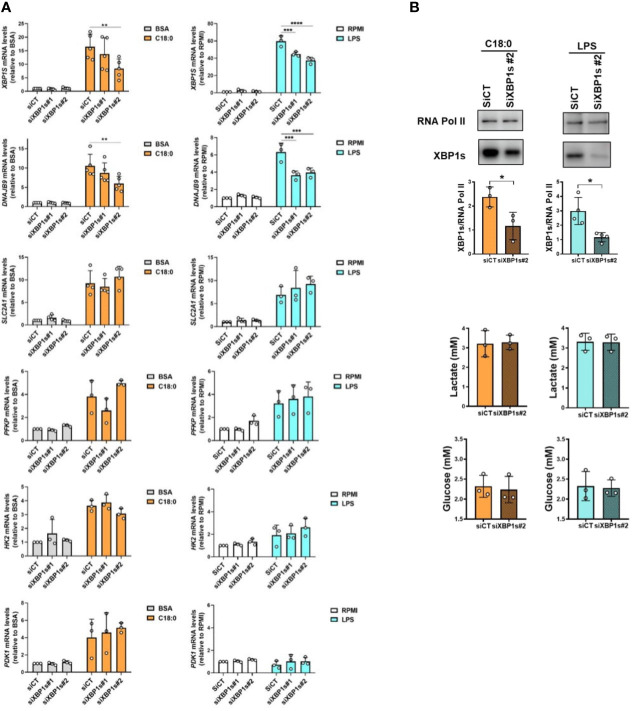
XBP1s is dispensable in C18:0- and LPS-mediated glycolysis activation. MDMs were transfected with siRNA control (siCT) or siRNA targeting XBP1s (siXBP1s#1 or siXBP1s#2) 24h before treating them or not (RPMI) with BSA, C18:0 (100 µM) or LPS (10 ng/ml). **(A)** After treatment for 24 hours with BSA or C18:0 and 3 hours with LPS, *XBP1S, DNAJB9, SLC2A1, PFKP, HK2* and *PDK1* mRNA levels were analysed by qRT-PCR. Two-Way ANOVA, Tukey’s multiple comparisons test, ****p< 0.0001, ***p< 0.001, **p<0.01. **(B)** Top panel: After 10h or 5h of treatment with C18:0 or LPS, respectively, XBP1s and RNA polymerase II protein levels were analysed by Western blotting on nuclear extracts. Unpaired t-test, *p<0.05. Bottom panel: After 24h of treatment with C18:0 or LPS, glucose and lactate extracellular concentrations were measured by NMR. Unpaired t-test.

The next step was to study the involvement of the transcription factor XBP1s in the activation of glycolysis in macrophages in response to both inducers. First, we studied the impact of XBP-1 silencing using two different siRNAs on glycolytic genes expression (**Figure 6A**). The XBP1s knockdown was checked by monitoring XBP1S mRNA levels through qRT-PCR assay. For C18:0 treatment, we can see a partial but significant depletion of XBP1S mRNA with siRNA #2 but not with siRNA #1 (**Figure 6A**). To ensure that this level of *XBP1S* mRNA depletion is sufficient to affect the expression of a XBP1s target gene, we measured the expression of the *DNAJB9* gene encoding the chaperone ERdj4 (45). As expected, the decrease in *XBP1S* mRNA levels is accompanied by a significant decrease in the expression of the *DNAJB9* gene but not of the glycolytic genes *SLC2A1, PFKP, HK2* and *PDK1* (**Figure 6A**). We observed a very significant increase in *XBP1S* mRNA levels after 3 hours of treatment with LPS (**Figure 6A**). These levels were partially but significantly downregulated with both siRNAs #1 and #2 (**Figure 6A**). Again, as expected, silencing of XBP1s induced a significant decrease in the induction of the *DNAJB9* gene but not of the glycolytic genes (**Figure 6A**). It should be noted that 3 hours of treatment with LPS was not enough to observe an upregulation of both *HK2* and *PDK1* genes; indeed, their induction requires a slightly longer treatment time (**Figure 2A**). XBP1s silencing, also confirmed by the decrease in XBP1s protein levels in nuclear extracts (**Figure 6B**, top panels), has no impact on lactate production and glucose consumption either (**Figure 6B**, bottom panels). Altogether, these experiments suggest that XBP1s is dispensable in both C18:0- and LPS-induced glycolytic switch.

### IRE1’s RNase activity is involved in both C18:0- and LPS-mediated HIF-1α activation through XBP1s-independent way

**Figure 7:**
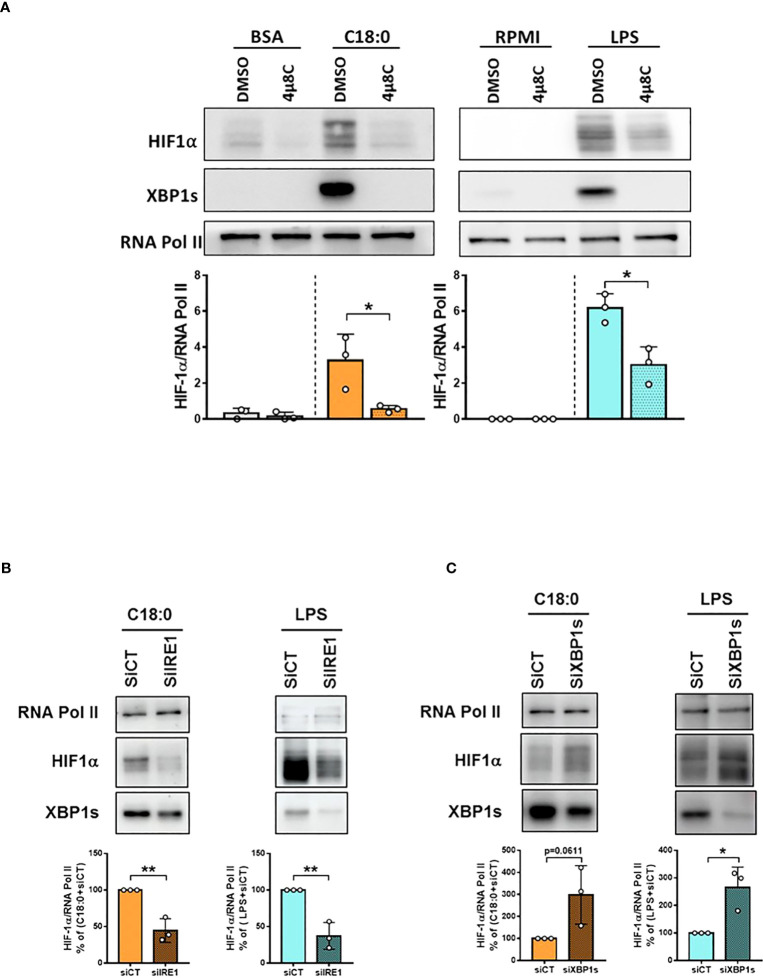
IRE1’s RNase activity is involved in C18:0- and LPS-induced HIF-1α activation through XBP1s-independent way. MDMs were pre-treated with DMSO or 4µ8C (50µM) for 1h **(A)** or transfected with siRNA control (CT) or siRNA targeting IRE1α for 48h **(B)** or siRNA targeting XBP1s for 24h **(C)** before treating them or not (RPMI) with BSA (10h), C18:0 (100 µM, 10h) or LPS (10 ng/ml, 5h). HIF-1α, XBP1s and RNA polymerase II protein levels were analysed by Western blotting on nuclear extracts. Unpaired t-test, *p<0.05, **p<0.01.

Since XBP1s is dispensable, we dropped the idea of an interaction between both transcription factors HIF-1α and XBP1s. Therefore, the impact of IRE1’s RNase inhibition was studied directly on HIF-1α activation. IRE1 inhibition by 4µ8C, as confirmed by the lack of XBP1s in the nuclei of C18:0- and LPS-treated macrophages, significantly impairs the accumulation of HIF-1α in the nuclei (**Figure 7A**). On the other hand, no effect of either 4μ8C or STF is observed on *HIF1A* mRNA levels (**Figure S3**). Interestingly, while both IRE1α and XBP1s knockdown induce a decrease in nuclear XBP1s levels, yet they have opposite effects on HIF-1α activation; knockdown of IRE1α inhibits while that of XBP1s promotes HIF-1α activation (**Figures 7B, C**). These results demonstrate that XBP1s is absolutely not required for the activation of HIF-1α. Another piece of information derives from these experiences; since XBP1s deficiency was shown to lead to a feedback hyperactivation of IRE1α and RIDD (46, 47), these results suggest that the activation of HIF-1α would involve RIDD activity.

## Discussion

A chronic low-grade inflammation, also called metaflammation, contributes to the pathological development of obesity (4, 5). This inflammation originates in the visceral adipose tissue undergoing significant remodeling characterized by both adipocyte hyperplasia and hypertrophy and a significant recruitment of immune cells. In this complex microenvironment enriched with nutrients (glucose, FFA), hormones (insulin), adipokines, the macrophages acquire a ‘metabolically activated’ phenotype with pro-inflammatory properties (6). SFAs released in excess from adipocyte lipolysis and cell death in obese adipose tissue have been proposed as triggers in shaping this ATM phenotype (6).

In order to better understand the mechanisms underlying this specific polarization, we sequenced the whole transcriptome of human macrophages treated with SFA. As expected, GSEA highlighted the pro-inflammatory profile at the top of the list. This analysis also revealed metabolic signatures like glycolysis and hypoxia. We confirmed the activation of both HIF-1α and aerobic glycolysis in SFAs-treated MDMs and demonstrated that this glycolytic switch supports the proinflammatory polarization. Since UPR is a prominent signature in this macrophage model and mediates most of SFAs effects, we tested its involvement in HIF-1α-dependent glycolytic switch. In this work, we demonstrate a role of IRE1’s RNase activity in HIF-1α activation driving the glycolytic switch in SFAs-activated macrophages. Interestingly, IRE1 fulfills this novel function independently on XBP1s.

LPS was used in each experiment as a M1 polarization inducer. Although this TLR4 agonist does not drive classical ER stress and UPR, it does induce specific activation of IRE1 by promoting its auto-phosphorylation through TRAF6-dependent mechanism (29). Interestingly, we also observed in these macrophages challenged with LPS a role of IRE1’s RNase activity in the activation of both HIF-1α and glycolysis supporting pro-inflammatory polarization. Again, XBP1s was dispensable. This novel function of IRE1 RNase in immunometabolic activation of macrophages may contribute to the development of metabolic inflammation in response to higher SFAs concentrations and/or to abnormal levels of circulating LPS resulting from increased intestinal permeability in obesity (48). This work also suggests such a role for IRE1 RNase in the pro-inflammatory response following infection by exogenous pathogens activating TLR4 or other pathogen recognition receptors (PRRs).

To our knowledge, these results are the first to demonstrate a role of IRE1, independent of XBP1s, in the activation of HIF-1α and glycolysis within pro-inflammatory macrophages. In patients with Cystic Fibrosis (CF), a recessive genetic disorder caused by mutations in the cystic fibrosis transmembrane conductance regulator (CFTR), accumulation of the misfolded CFTR induces a perpetual ER stress (49). An overactive IRE1α-XBP1 pathway reprograms CF M1 macrophages toward an increased metabolic state with increased glycolytic rates and mitochondrial function, associated with exaggerated production of TNFα and IL-6. This hyper-metabolic and -inflammatory state, seen in CF macrophages, is reversed by inhibiting the RNase domain of IRE1α (49). However, a possible role of HIF-1α is not mentioned. More recently, a role of IRE1’s RNase activity was demonstrated in upregulation of both *HIF1A* mRNA and glycolysis in macrophages following *B. abortus* infection but this pathway relies on XBP1s (50). Only one report demonstrated a link between IRE1’s RNase activity and HIF-1α activation through XBP1s-independent mechanism in endothelial cells under hypoxia (51).

Although IRE1’s RNase activity is required for HIF-1α activation by both LPS and SFAs, underlying mechanisms and kinetics are quite different. LPS-mediated IRE1 activation is rapid, as demonstrated by both *XBP1S* mRNA and XBP1s protein peaking at 3 h. Furthermore, the XBP1s protein is no longer visible beyond 5 hours of activation by LPS, demonstrating a more transient activation of IRE1 than with the classic inducers, Tm and Tg (52). This particular kinetics may be related to a two-wave modulation of IRE1 protein levels in macrophages after LPS challenge; indeed, IRE1 levels seem to increase very transiently at short times (3h), then decrease before increasing again at long times (16-24h). The activation of IRE1 in response to C18:0 results from phospholipid saturation and lipid bilayer stress (18, 19). Accordingly, the kinetics are slower and sustained over time since the *XBP1S* mRNA accumulates for up to 24 h. Similarly, the XBP1s protein shows a peak at 10h but is still visible at 24h. This kinetics is consistent with the progressive accumulation of IRE1 protein in macrophages upon activation with C18:0. Such increase in IRE1 levels has been also observed in response to classical ER stress inducers in several cell lines and has been shown to result, at least partially, from JNK-dependent transcriptional activation (52). However, accumulation of IRE1 protein during ER stress was also reported to be caused by attenuation of BiP-dependent degradation of IRE1 (53). Further investigation will be required to resolve the origin of these IRE1 protein modulations in macrophages upon LPS or SFA stimulation.

Anyway, the kinetics of IRE1 activation, although different for each inducer, match perfectly with those of HIF-1α activation and glycolytic genes expression. We also observed a very significant upregulation of IRE1 protein levels in C18:0- and LPS-treated macrophages when HIF-1α was knocked down (data not shown), which looks like to a feedback during which the cell compensates for the loss of HIF-1α by upregulating IRE1 levels. Taken together, these observations further strengthen our results demonstrating a crosstalk between both pathways, IRE1 and HIF-1α.

Since XBP1s is dispensable, IRE1 RNase should fulfill this novel function through RIDD. Such mechanism suggests the involvement of a mRNA or pre-miRNA target which would be cleaved by IRE1 endonuclease in the consensus sequence 5′-CUG↓CAG located within a stem-loop secondary structure similar to the one observed in *XBP1S* mRNA and would be subsequently rapidly degraded by cellular exoribonucleases (20–23). RIDD regulates several additional cellular functions besides reducing ER load, including triglyceride and cholesterol metabolism (54), apoptosis signaling (55), protective autophagy (56) and DNA repair (57). Several dozen RIDD mRNA targets have been highlighted so far but these may depend on the nature of the stress stimuli and tissue and cell context (58). Many RIDD targets are yet to be identified. 

Interestingly, we noted that the XBP1s knockdown, that is known to induce hyperactivation of IRE1 and increased RIDD activity (46, 47), further increases HIF-1α activation in C18:0- and LPS-treated macrophages. These observations support the hypothesis involving RIDD as the missing link between IRE1 and HIF-1α. What could be the mRNA or pre-miRNA targeted by RIDD whose degradation favors the accumulation of HIF-1α? Given the complexity of the multilayer HIF-1α regulation, the possibilities are multiple. The nonhypoxic stabilization of HIF-1α in LPS-activated macrophages is mediated by tricarboxylic acid (TCA) cycle intermediates like succinate and citrate that accumulate following LPS treatment in macrophages and lead to PHD inhibition through various mechanisms, notably through the production of reactive oxygen species (ROS) (41). Since the transcriptomic analysis revealed a ROS signature (**Figure 1C**) and the modulation of some TCA cycle enzymes (data not shown) in C18 :0-treated MDMs, we can suggest the involvement of such mechanism in C18 :0-mediated HIF-1α activation. 

IRE1α activation has been frequently observed in multiple tissues and cell types from dietary and genetic obesity mouse models (26, 28). Hyperactivation of the IRE1α–XBP1 pathway has been also documented in adipose tissue of obese humans (59, 60). This state of so-called metabolic ER stress is provoked by a multitude of stimuli, mainly nutrients like glucose, lipids,… arising from both systemic and tissue microenvironmental changes in the face of energy surplus (28). Chronic IRE1 activation under such metabolic ER stress contributes to the pathological progression of obesity by disrupting some metabolic and inflammatory pathways in tissues like liver and pancreatic islets (28). While XBP1s can be viewed as an adaptive effector in the homeostatic control of metabolism, it has been increasingly recognized that hyperactivation of IRE1’s RIDD activity can mediate its maladaptive, pathological effects during obesity-induced metabolic stress (28).

Interestingly, IRE1α was shown to be also activated in ATMs of DIO mice (61). Myeloid-specific IRE1α abrogation in mice blocked high fat diet (HFD)-induced obesity and insulin resistance and reversed HFD-induced M1–M2 imbalance in white adipose tissue (WAT) (61). To define the role of IRE1α in shaping the inflammatory properties of macrophages, Shan et al. (61) stimulated BMDMs with LPS or IL4 to mimic M1 or M2 polarization *in vitro*. They demonstrated a role of IRE1α both in upregulation of M1 markers such as iNOS and IL-6 and in downregulation of some M2 markers and proposed XBP1s- and RIDD-dependent mechanisms, respectively. Our results fit with those of Shan et al. since they also demonstrate a role of IRE1 RNAse in the M1-like polarization of human macrophages. The mechanism highlighted in our work is different but complementary since it involves IRE1’s RNase activity, but not XBP1s, in the HIF-1α-mediated glycolytic switch required for the M1-like polarization of macrophages. This mechanism probably relies on RIDD. 

Although activation of glycolysis controlling cytokine release has been validated in ATMs of DIO mice (62), a limitation of this work is that it does not allow to confirm the causal link between IRE1α activation and glycolysis stimulation *in vivo*. Comparing the transcriptome of ATMs from WT mice with myeloid-specific IRE1α deficient mice after a normal chow or high-fat diet would allow us to confirm or not the involvement of IRE1α in the activation of glycolysis *in vivo*. This analysis could also highlight genes that are downregulated by HFD in an IRE1α-dependent manner and that constitute potential RIDD targets. These relevant RIDD substrates would be selected for a functional study in the model of BMDMs (or MDMs) + SFA (or LPS). The best RIDD candidates could be the target of new therapeutic strategies that would have the advantage of not impacting the production of XBP1s that functions rather to protect metabolic tissues in obesity (28).

## Data availability statement

The original contributions presented in the study are publicly available. This data can be found here: [https://www.ncbi.nlm.nih.gov/geo/query/acc.cgi?acc=GSE233446].

## Author contributions

MI performed most of the experiments, collected and analyzed the research data. MCo performed MDMs differentiation and treatments for RNAseq as well as data analyses. CW, MCa, CL, OP, and PT contributed to technical assistance. MI, MCo, LL, JP, and SL-P conceived and designed the experiments. SL-P wrote the manuscript. All authors revised and approved the manuscript.
