# Supplementary material for: Novel XBP1s-independent function of IRE1 RNase in HIF-1α-mediated glycolysis upregulation in human macrophages upon stimulation with LPS or saturated fatty acid
Source: Front Immunol. 2023 Aug 30;14:1204126. doi: 10.3389/fimmu.2023.1204126 (PMC10498766; doi:10.3389/fimmu.2023.1204126)
Supplement: Supplementary file 1 [file DataSheet_1.pdf]

SUPPLEMENTARY DATA

Table S1 : Human qPCR primers

	Forward	Reverse
GLUT1	CTGGCATCAACGCTGTCTTCTATTAC	CCACCACAAACAGCGACACG
GLUT3	ATGGGGACACAGAAGGTCAC	CCTTTATGATCTTCTCAGGAGCATTG
HK2	AACTAGACGAGAGTTTCCTGGTC	CGATATCAAAGTCCCCTCTCCTC
PFKP	CAAACTCTCGGAGAACCGTGC	GCGTGACGACAAGCTCTTTG
PFKFB3	ATTGCGGTTTTCGATGCCAC	GCCACAACTGTAGGGTCGT
ALDOA	GCACCGGAACTTGCTACTACC	GGCAATGCTCCCAGTGGAC
PKM1	GTGAGCAGACCTGCCAGACT	CGAGCCTCAAGTCACTCCAC
PKM2	ATTATTTGAGGAACTCCGCCGCCT	ATTCCGGGTCACAGCAATGATGG
LDHA	GGAGATTCCAGTGTGCCTGTATG	CCTCATAAGCACTCTCCACCACC
PDK1	AGTGCCTCTGGCTGGTTTTG	GCATCTGTCCCGTAACCCTC
XBP1u	TGGTTCCTGAAGAGGAGGCGGAAG	GAGATGTTCTGGAGGGGTGACAACTG
XBP1s	CTGAGTCCGCAGCAGGTG	ACTGGGTCCAAGTTGTCCAG
DNAJB9	TGGTGGTTCCAGTAGACAAAGG	CTTCGTTGAGTGACAGTCCTGC
TBPH	ACAGCCTGCCACCTTACG	TGCCATAAGGCATCATTGGACTA

Table S2 : siRNA sequences

siRNA	Forward	Reverse
XBP1s #1	5’-UAGAAAAUCAGCUUUUACGAGAGAA-3’	5’-UUCUCUCGUAAAAGCUGAUUUUCUAGC-3’
XBP1s #2	5’-CAACUUGGACCCAGUCAUGUUCUTC-3’	5’-GAAGAACAUGACUGGGUCCAAGUUGUC-3’
IRE1α #1	5’-GGACAGGCUCAAUCAAAUGGACUUU55-3’	5’-AAAGUCCAUUUGAUUGAGCCUGUCC55-3’
IRE1α #2	5’-CCGAAGUUCAGAUGGAAUCCUCUAC55-3’	5’-GUAGAGGAUUCCAUCUGAACUUCGG55-3’
HIF-1α #1	5’-UCACCAAAGUUGAAUCAGAAGAUAC-3’	5’-GUAUCUUCUGAUUCAACUUUGGUGAAU-3’
HIF-1α #2	5’-CGGUUGAAUCUUCAGAUAUGAAAAT-3’	5’-AUUUUCAUAUCUGAAGAUUCAACCGGU-3’
Negative Control	5’-CGUUAAUCGCGUAUAAUACGCGUAT-3’	5’-AUACGCGUAUUAUACGCGAUUAACGAC-3’

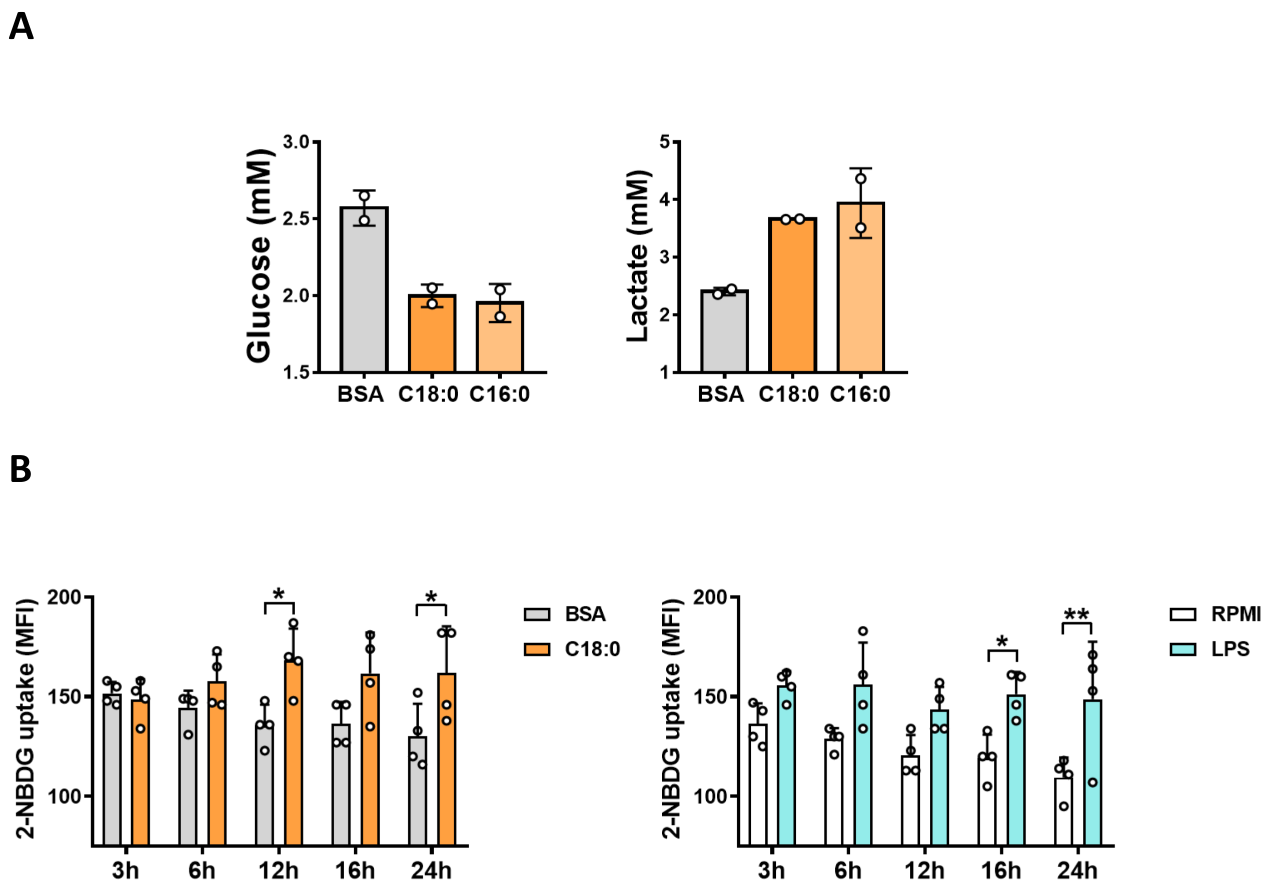

FIGURE S1. (A) C16:0 (palmitate) also induces glycolysis activation in human macrophages. Glucose and lactate concentrations were measured by NMR in supernatants of MDMs treated with C18:0 (100 µM), C16:0 (100 µM) and BSA for 24h (two independent experiments). Data are mean ± SD. **(B)** Glucose uptake by MDMs treated with BSA, C18:0 (100 µM) or cultured with LPS (10 ng/ml) and medium alone (RPMI) for indicated times was determined by the fluorescence of 2-NBDG (n=4). Data are mean ± SD. 2way ANOVA, Sidak's multiple comparisons test, *$p < 0.05$, **$p < 0.01$.

A

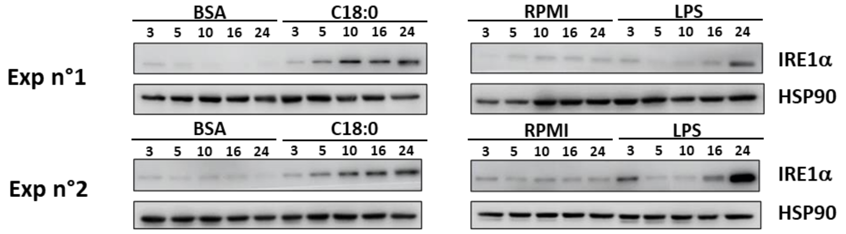

B

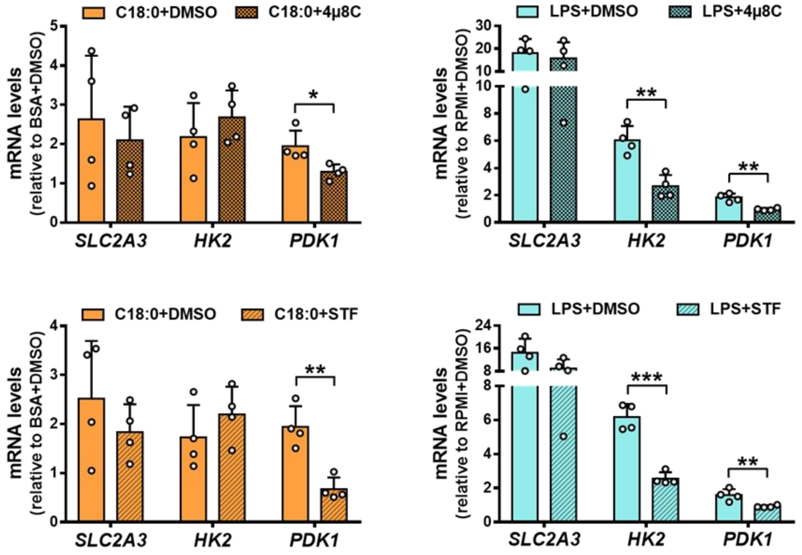

FIGURE S2: **Involvement of IRE1α in C18 :0- and LPS-mediated glycolysis activation. (A)** IRE1α and HSP90 protein levels were analysed by Western blotting in total extract of MDMs treated with BSA or C18 :0 (100 µM), maintained in medium alone (RPMI) or stimulated with LPS (10 ng/ml) for indicated times. Two representative experiments (Exp n°1 and Exp n°2). **(B)** MDMs were pre-treated for 1h with STF (100 µM), 4µ8C (50 µM) or vehicule (DMSO) before the addition or not (RPMI) of BSA and C18 :0 (100 µM) for 24h or LPS (10 ng/ml) for 6h. qRT-PCR analysis of *SLC2A3*, *HK2* and *PDK1* mRNA levels. Unpaired t-test , ***p< 0,001, **p<0,01, *p<0,05.

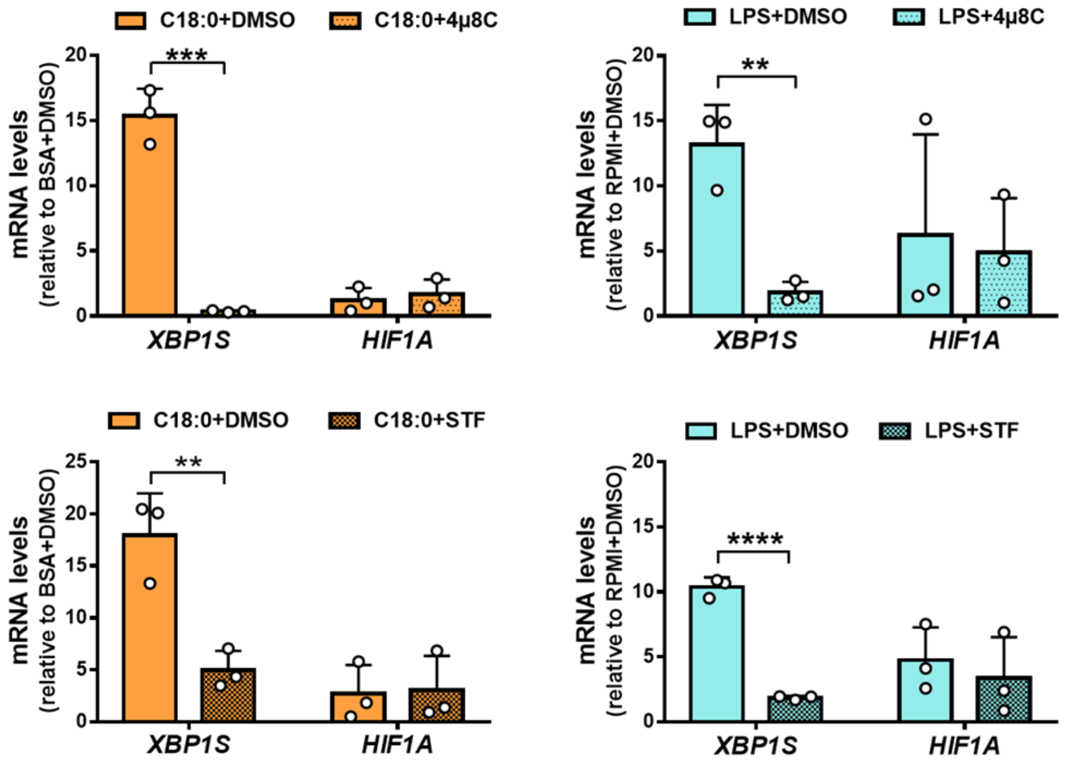

FIGURE S3: *HIF1A* mRNA levels are not affected by IRE1α's RNAse activity inhibition. MDMs were pre-treated for 1h with STF (100 µM), 4µ8C (50 µM) or vehicule (DMSO) before the addition of C18 :0 (100 µM) for 24h or LPS (10 ng/ml) for 8h. *XBP1S and HIF1A* mRNA levels were analysed by qRT-PCR. Unpaired t-test , ****p<0.0001, ***p< 0,001, **p<0,01, *p<0,05.
